# Impact of a pediatric primary care health-coaching program on change in health-related quality of life in children with mental health problems: results of the PrimA-QuO cohort study

**DOI:** 10.1186/s12875-023-02119-0

**Published:** 2023-09-08

**Authors:** Verena Loidl, Karina Hamacher, Martin Lang, Otto Laub, Lars Schwettmann, Eva Grill

**Affiliations:** 1 Institute for Medical Information Processing, Biometry, and Epidemiology (IBE), Faculty of Medicine, LMU Munich, Munich, Germany; 2 Pettenkofer School of Public Health, Munich, Germany; 3 BKK Vertragsarbeitsgemeinschaft Bayern, Munich, Germany; 4 PaedNetz Bayern e.V., Munich, Germany; 5 Berufsverband der Kinder- und Jugendärzte (BVKJ) e.V., Cologne, Germany; 6 Helmholtz Zentrum München (GmbH) – German Research Center for Environmental Health, Institute of Health Economics and Health Care Management (IGM), Neuherberg, Germany; 7 Department of Economics, Martin Luther University Halle-Wittenberg, Halle (Saale), Germany; 8 German Centre for Vertigo and Balance Disorders, LMU University Hospital, LMU Munich, Munich, Germany; 9 Munich Center of Health Sciences, Ludwig-Maximilians-Universität München, Munich, Germany

**Keywords:** Health care service, Children and adolescents, KINDL-R, Health-related quality of life

## Abstract

Mental health problems (MHP) have a considerable negative impact on health-related quality of life (HRQoL) in children and their families. A low threshold Health Coaching (HC) program has been introduced to bring MH services to primary care and strengthen the role of pediatricians. It comprised training concepts as a hands-on approach for pediatricians, standardization of diagnosis and treatment, and extended consultations. The aim of this study was to evaluate the potential effects of the HC on HRQoL in children with MHP and their parents.

We used data from the PrimA-QuO cohort study conducted in Bavaria, Germany from November 2018 until November 2019, with two assessments one year apart. We included children aged 17 years or younger with developmental disorder of speech and language, non-organic enuresis, head and abdominal pain, and conduct disorder. All included children were already part of the Starke Kids (SK) program, a more general preventive care program, which includes additional developmental check-ups for children enrolled in the program. In addition,  treatment according to the HC guidelines can be offered to children and adolescents with mental health problems, who are already enrolled in the SK program. These children form the intervention group; while all others (members of BKK and SK but not HC) served as controls. HRQoL in children was assessed using the KINDL questionnaire. Parental HRQoL was measured by the visual analogue scale. To analyze the effects of the intervention on children´s HRQoL over the 1-year follow-up period, we used linear mixed effects models.

We compared 342 children receiving HC with 767 control patients. We could not detect any effects of the HC on HRQoL in children and their parents. This may be attributed to the relatively high levels of children´s HRQoL at baseline, or because of highly motivated pediatricians for the controls because of the selection of only participant within the Starke Kids program. Generally, HRQoL was lower in older children (-0.42 points; 95% CI [-0.73; -0.11]) and in boys (-1.73 points; 95% CI [-3.11; -0.36]) when reported by proxy. Parental HRQoL improved significantly over time (2.59 points; 95% CI [1.29; 3.88]).

Although this study was not able to quantitatively verify the positive impact of this HC that had been reported by a qualitative study with parents and other stakeholders, and a cost-effectiveness study, the approach of the HC may still be valid and improve health care of children with MHP and should be evaluated in a more general population.

**Supplementary Information:**

The online version contains supplementary material available at 10.1186/s12875-023-02119-0.

## Introduction

Mental health problems (MHP) have a considerable negative impact on health-related quality of life (HRQoL) in children and their families [1, 2]. Worldwide, it is estimated that 13% to 20% of children and adolescents suffer from MHP [3, 4]. In Germany, overall prevalence of MHP is stable on a high level [5], with over 17% of children and adolescents showing clinically relevant MHP [6]. Among these, developmental disorders (17%), followed by conduct disorders (11%) are the most frequent conditions [5, 7]. In addition, it has been shown, that the risk of chronification and persistence of MHPs in adulthood increases, when symptoms of MHP occur during childhood or adolescence [8–12]. To give an example, a German national cohort study has shown that externalizing as well as internalizing problems in childhood or adolescence are associated with poorer general mental health and a higher incidence of depressive symptoms, and a higher risk to suffer from eating disorder symptoms in adulthood [13].

Structural problems of the healthcare system such as a lack of intersectoral coordination – a complex approach, which is integrated across different health care sectors – timely access to care and adequate standardization of diagnostics and treatment have been mentioned as the main obstacles to adequate management of youth MHP in Germany [14]. In the German statutory health insurance (SHI) system, children are insured along with their parents without any additional charges. The SHI covers most of the costs associated with children's healthcare needs, including mental health care. Specifically, primary care has been mentioned as one key sector for early recognition and timely treatment of MHP in children and adolescents [15]. Primary care in Germany is provided by practices run by independent specialists (e.g. pediatricians (PD)) who offer services to patients under the statutory health insurance scheme. This is mandatory for the majority of the population. Within the framework of statutory health insurance, specialists in pediatrics can only treat children and adolescents up to the age of 18 and they can be consulted without any registration, gatekeeping, or referrals. Pediatricians are seeing children on a regular basis for routine checks and might therefore recognize mental health (MH) needs at an early stage [16, 17]. Typically, the PD would perform an initial screening, initiate treatment and recommend referral to specialized centers in severe cases. However, due to time constraints in daily practice and a potential lack of specific MHP expertise patient needs may not be addressed adequately [18]. It has been noted that referrals to specialized care tend to be the standard approach, irrespective of the severity of the problems, causing bottlenecks for those who need specialized care [19].

Against this background, a targeted but low-threshold MH primary care program – Health Coaching (HC) – was developed and implemented by a group of statutory health insurance funds (Betriebskrankenkassen Landesverband Bayern, BKK-LV [20]) in collaboration with pediatricians [21] in 2011 [22–24]. The BKK-LV – an umbrella organization for all BKKs health insurance funds in Bavaria (17 members) – is one of the biggest statutory health insurance companies in Germany and was involved in the development of the HC for children and adolescents with MHP. As all children and adolescents, participating in the study, were insured at the BKK funds no private funding was spent. The BKK is a major statutory health insurance funds in Germany with 10.9 (in Bavaria: 2.5:) of a total of 73.0 million insures. HC provides standardized and evidence-based diagnostic and management guidelines for 16 MH conditions, which are taught in aspecific training for PD. Participating PDs get familiarized with the use of the standardized guidelines to improve detection and treatment of MHP. They thus are supported in their decision-making process to decide if the child can be treated in primary care or – for severe cases – whether an immediately referral to a specialized care provider is necessary. The treatment guidelines of the HC also have the potential to counteract a misuse of medication in cases where non-pharmacological treatment is more appropriate. If pharmacological treatment is needed, the child will be referred to specialized care in order to receive the optimal pharmacological treatment. Moreover, standardized guidelines for actions support PDs to perform standardized treatment and the integration of person- and environment-related factors of the children and their families (e.g. better self-management skills, extended resources like care services available, or resilience factors. PDs receive an additional reimbursement from the health insurance fund for every child or adolescent inscribed into the program and treated according the HC specifications (additional amount of 15 euros per 10 min up to a cap of 180 min). While this is a promising approach to avoid overtreatment and misuse of medicalization there is a lack of evidence regarding its effectiveness.

Evidence of the benefits of integrating MH interventions in primary pediatric care is still weak. There is an example from the Netherlands of an effective program where pediatricians have been trained successfully in delivering MH services. They found an increased identification rate for MHP, more doctor´s visits because of MHP and less psychopharmacological prescriptions been issued [25]. Also countries like the UK [26], Australia [27, 28], and Canada [29] have already made successful steps to integrating MH services into primary pediatric care. In recent years, there has been growing interest to include patient-reported outcome measures in child and youth MH settings [30]. In this context HRQoL of children suffering from MHP is an essential outcome, because it shows the direct (e.g. change of behavioral problems) as well as the indirect (e.g. change in dealing with peers) effects of the HC [31]. HRQoL measures allow for a patient-centered approach to healthcare. By assessing the impact of the HC on childrens' daily lives, HRQoL measures provide a more comprehensive understanding of the benefits and limitations of the HC from the patient's perspective. Furthermore, the HC is complex and may have effects which go beyond the improvement of behavior problems and social skills. HRQoL measures can help to capture these diverse effects of the HC, since it also includes changes in physical function, psychological well-being, or social support by family and friends.

The aim of the study was to investigate the potential effects of the Health Coaching (HC) program [22–24] on health-related quality of life of children and adolescents with MHP and their parents compared to those children and adolescents with MHP and their parents, who did not receive the HC.

## Methods

### Study design

The prospective PrimA-QuO cohort study was conducted in Bavaria, Germany with measurements at two time points one year apart (baseline: from January to November 2018; follow-up: from January to November 2019). The collection of data was performed using an online questionnaire.

### Participants

The population comprised children and adolescents aged 0 to 17 years with developmental disorder of speech and language (SLD), non-organic enuresis (NE), head and abdominal pain, somatoform (HAP) and conduct disorder (CD). Diagnoses were identified using the International Classification of Diseases diagnosis codes (ICD-10 [32]) namely: SLD: F80.0-F80.9; NE: F98.0; HAP: G44.2, G43.0, G43.1, R10.4, F45.4; CD: F68.8, F91.0-F92.9, F94.0-F95.9, F98.3-F98.9. All children were insured at a BKK SHI. The roof organization of the BKK SHIs is the BKK Landesverband Bayern (LV), with 2.5 million insures [33].

All children were insured at the BKK funds, had been enrolled in the BKK Starke Kids (SK) program, a health promotion program, which offers additional developmental check-ups for children and adolescents [34]. The program is offered to BKK-insured families free of charge and is available nationwide. It is part of the BKK's broader commitment to promoting health and wellbeing among its members and the wider community. Children have to be enrolled by their parents in this program. All children had at least one consultation for succeeding diagnoses at an office-based pediatrician in Bavaria, Germany, from July 2017 to November 2018. The identification of eligible children was based on billing data. As billing data were available with a delay of up to six months, the time point of enrollment of the child in the HC can only be approximately determined. Parents of eligible children, found in the BKK insurance database, were contacted by the BKK health fund via mail and provided with a link to the questionnaire. Survey data were collected online using SoSci Survey [35]. Access was regulated by users’ authentication via their insurance number. The link for the follow-up questionnaire was provided by the study team via email one year after baseline. Families received a small monetary compensation for participation.

Children in the intervention group participated in the SK program and were treated by a pediatrician trained in the HC [22] – comprising standardized and evidence-based diagnostic and management guidelines and specific training for pediatricians (IG: members of BKK, SK, and HC). Children in the control group were members of the BKK, but not necessarily enrolled in the SK program (CG: members of BKK, SK or not SK, but not HC). The diagnostic and treatment guidelines were specifically developed and target for the diagnoses groups.There was confinement to subjects with complete data. Detailed information on the study design were published elsewhere [22]. We included 1109 children and adolescents and obtained a response rate of 17% at baseline (7.343 invitation letters sent) and 56% at follow-up (998 invitation letters sent) with regard to questionnaires. This response rate is not atypical when participants with specific diagnoses are identified from a health insurance database, which has several advantages, for instance the BKK health insurance database contains a large number of patients, which provides a larger potential sample size than traditional recruitment methods, and patients are not limited to specific geographical areas or health care institutions. Furthermore, it is an efficient method compared to other recruitment methods as potential patients with a specific diagnosis can be identified, which is especially useful for children with MHP because they are usually not treated by a pediatrician.

Approval had been obtained from the local ethics committee (approval number 17–497) and the Data Protection Officer of the Ludwig-Maximilians-University Munich. All procedures were designed in full compliance with European and national data protection legislation [36, 37]. Informed consent was elicited from the parents and from children/adolescents aged six or older. Participants received age-appropriate and detailed information regarding the background and implementation of the study. They were offered the opportunity to revoke their participation in the study at any time.

### Measures and instruments

Primary outcome was the health-related quality of life (HRQoL) of children. We used the generic and validated German-language instrument *KINDL*^*R *^(Kinder-Lebensqualitätsfragebogen) [38]. It consists of 24 items divided between six dimensions (with four items each) with reference to the past week: physical well-being, emotional well-being, self-worth, well-being in the family, well-being related to friends/peers, and school-related well-being. Each item provides answer on a five-point Likert scale ranging from “never” to “always” coded with values between 1 and 5. The higher values indicating "better" HRQoL ratings. The total HRQoL score was calculated for all 24 items. The item scores per dimension (and the total score) were added and transformed into values between 0 and 100 (total sum = total mean * 24; total score = ((total sum – 24)/96)*100). The child and adolescent self-assessment version was used for children aged eleven years or older at baseline; for younger children the proxy version was completed by the parents. As several studies suggest, parental and self-assessment of the KINDL^R ^total score were reported separately for subsequent analyses [39, 40]. The KINDL questionnaire revealed good scale properties in terms of floor and ceiling effects as well as scale fit. In terms of reliability, the subscales showed moderate internal consistency [41]. In chronically ill population the psychometric properties appeared to be somewhat higher [42].

Secondary outcome was parental HRQoL of affected children, measured by the EQ-5D visual analogue scale (VAS) that records self-rated overall health state (range 0 to 100, with higher scores indicating better quality of life [43].

Sociodemographic information, namely age, and sex of the child, age, sex, and educational level of both parents, and disease related data, namely MHD group diagnosis were collected at baseline. Parental education was grouped into three categories: low (no formal qualification and secondary school), medium (intermediate school, no high school graduation) and high (high school or university graduation).

### Statistical analyses

Descriptive statistics for categorical and continuous variables were expressed as percentages and means. Bivariate non-parametric tests (Mann-Whitney-U test, Chi-squared test) were used to test for differences between the intervention and control group at baseline.

Because children were not randomly assigned to receive HC or standard care, a propensity score-weighted analysis [44] was performed to reduce the effect of selection bias and simulate the effects of randomization. Propensity scores (the conditional probabilities of receiving HC or not given the observed covariates) were estimated using a non-parsimonious multiple logistic regression model based on age (continuous variable), sex, educational level of the parents and diagnoses group (categorical variables) at baseline. Data were weighted with the inverse probability of treatment weighting (IPTW) method [45], using stabilized weights. Covariate balance, indicating adequacy of the propensity score model specification, was checked with standardized differences (absolute values < 0.1 supported the assumption of balance between groups) [44].

To analyze the effects of intervention on children´s HRQoL over the 1-year follow-up period, we used linear mixed effects models with subject-specific random intercept. The continuous outcome of the models was the KINDL total score. Subsequently all KINDL subscale scores were used as outcome. When conducting the linear mixed effects models for the HRQoL of the children, we used the respective highest parental educational level as mothers’ and fathers’ educational levels were highly correlated. To account for potential differences between the intervention and control groups, all models were controlled for sex of the child and highest educational level of the parents as well as diagnoses group and intervention, which were introduced in the model with dummy coding. Additionally, age of the child was introduced as continuous variable. Taking into account by-subject variability, we had intercepts for subjects as random effects. To observe group differences in their changes in HRQoL we included the interaction with time. Time was introduced as a fixed slope as the model fit was better presented assessed by the Akaike information criterion (AIC), whereas a lower AIC indicates a better fit.

To analyze the effects of intervention on parental HRQoL over the 1-year follow-up period, we used linear mixed effects models with subject-specific random intercept. The continuous outcome of the models was the VAS. To account for potential differences between parents, whose children were in the intervention or control group, sex and the educational level of the parent who had completed the questionnaire, as well as diagnoses group and intervention of the child were introduced in the model with dummy coding. Age of the parent and HRQoL of the child were introduced as continuous variables. Taking into account by-subject variability, we had intercepts for subjects as random effects. To observe group differences in their changes in HRQoL we included the interaction with time. Time was introduced as a random slope as the model fit was better presented.

Mixed effect models are widely applicable in longitudinal research as they allow to include participants with different numbers of measurement points, meaning that participants with incomplete data at follow-up can still be included in the analysis [46]. Overall model fit was assessed by the AIC, whereas a lower AIC indicates a better fit. In order to compare the AIC from the different models, each model must be based on the same participants. Therefore, the number of participants with complete observations regarding all covariables was included in the models.

Models were fitted using a restricted maximum likelihood approach (REML). Unadjusted and adjusted models were fitted. The local significance level was set at alpha 0.05. *P*-values were regarded noticeable in case *p* ≤ 0.05.

Plausibility checks were conducted before starting the analysis and deviations from homoscedasticity and normality were checked by visual inspection of residual plots. Analyses were performed using R version 4.0.3 [47, 48] and *nmle* [49] for linear mixed effects models.

## Results

### Study population

We included 1109 children and adolescents at the age of 0 to 17 years (40% female, mean age 6.9, SD 3.4). The total number of children receiving the intervention was 342 (31%). Table 1 shows the baseline characteristics of both groups. Groups were comparable regarding sociodemographic factors and HRQoL at baseline, with the exception of MHP diagnoses, which differed significantly between groups.

**Table 1:** Descriptive characteristics of the total cohort and by intervention group (HC) and control group (no HC) at baseline

Covariates	Total	No HC (Control)	HC (Intervention)	*p*-value*
*N*	*n* = 1109	*n* = 767	*n* = 342	
Age child [years]^a^	6.9 (SD = 3.4)	6.9 (SD = 3.4)	7.01 (SD = 3.3)	0.476
Girls^b^	446 (40%)	311 (41%)	135 (39%)	0.787
Age father [years]^a^	41.1 (SD = 6.1)	41.0 (SD = 6.1)	41.4 (SD = 6.2)	0.252
Age mother [years]^a^	38.1 (SD = 5.2)	38.0 (SD = 5.4)	38.3 (SD = 4.9)	0.506
**Highest educational level of both parents**^**b**^				
low	106 (10%)	73 (10%)	33 (10%)	0.821
medium	433 (39%)	304 (40%)	129 (38%)	
high	569 (51%)	389 (51%)	180 (53%)	
Diagnosis group child**^b^				
Head and abdominal pain, somatoform	227 (20%)	171 (22%)	56 (16%)	0.030
Developmental disorder of speech and language	582 (52%)	443 (58%)	139 (41%)	< 0.001
Conduct disorder	272 (25%)	168 (22%)	104 (30%)	0.003
Non-organic enuresis	96 (9%)	36 (5%)	60 (18%)	< 0.001
**Health-related quality of life**				
**KINDL-R parent proxy-report (*n* = 891)**				
KINDL-R total parent proxy report^a^	79.9 (10.7)	79.9 (10.6)	79.9 (11.1)	0.990
KINDL-R subscales parent proxy reports^a^				
Physical well-being	80.7 (17.1)	80.7 (17.0)	80.8 (17.3)	0.899
Emotional well-being	85.1 (13.1)	85.1 (12.9)	85 (13.6)	0.874
Self-worth	75 (14.2)	75.1 (14.3)	74.7 (14.1)	0.730
Well-being in the family	80 (13.9)	79.8 (13.9)	80.3 (13.9)	0.626
Well-being related to friends	79.8 (14.8)	79.9 (15)	79.4 (14.3)	0.647
School-related well-being	79 (17.1)	78.7 (17.1)	79.6 (17.2)	0.493
**KINDL-R child self-report (*n* = 163)**				
KINDL-R total child self-report^a^	72 (14.5)	71.6 (14.7)	72.9 (14)	0.581
KINDL-R subscales child self-reports^a^				
Physical well-being	72.3 (19.8)	72.9 (19.4)	70.9 (20.8)	0.547
Emotional well-being	75.9 (17.1)	75.3 (17.6)	77.7 (15.7)	0.417
Self-worth	64.3 (17.8)	63.6 (18.3)	66 (16.7)	0.439
Well-being in the family	77.8 (18.9)	76.8 (19.5)	80.2 (17.5)	0.306
Well-being related to friends	71.8 (20.2)	71.4 (21.0)	72.9 (18.1)	0.672
School-related well-being	69.4 (19.9)	69.1 (19.8)	70.1 (20.4)	0.770
**VAS parental health-related quality of life (*n* = 1083)**				
VAS^a^	84.4 (SD = 14.5)	84.2 (SD = 15)	84.8 (SD = 13.1)	0.528

*HC* Health Coaching. *VAS* Visual Analogue Scale

^*^P-value from Chi2-test for categorical variables and from Kruskal–Wallis test for continuous variables

^a^mean (SD: standard deviation)

^b^n: number (percentage: %)

Of all participants, 1054 completed the KINDL questionnaire (84.5% parent proxy-report) at baseline. Information for the KINDL during follow-up was available for 55.5% of the baseline participants. On average, the KINDL total score for the parent proxy-report version was 79.91 (SD 10.73) points at baseline and 79.16 (SD 10.73) points at follow-up. On average, the KINDL total score for the child self-report version was 71.95 (SD 14.51) points at baseline and 73.36 (SD 12.10) points at follow-up.

The VAS was completed by 1083 parents at baseline. Information for the VAS during follow-up was available for 56.4% of the baseline parents. On average, the VAS score was 84.39 (SD 14.50) points at baseline and 86.38 (SD 12.07) points at follow-up.

### Children´s health-related quality of life model

Results from the linear mixed effects models for the effect of the HC with children’s HRQoL are shown in Table 2. The model for the parent proxy-report was based on *n* = 891 and the model for the child self-report was based on *n* = 163 participants. No effect between the HC and children´s HRQoL total score was found after adjusting for age, sex, diagnosis group, and parental education for both models. For the parent proxy-version higher age of the child was significantly associated with lower HRQoL (-0.42 points; 95% CI [-0.73; -0.11]), as was male sex.

**Table 2:** Results of the linear mixed effects models with the KINDL total score parent proxy-report and KINDL total score child self-report as dependent variables controlled for time, age (in years), sex, educational level of the parents and diagnoses

**KINDL-R total**	**Parent proxy-report** (*n* = 891)			**Child self-report** (*n* = 163)		
	Estimate	95%-CI	*P*-value	Estimate	95%-CI	*P*-value
Intercept	82.36	[77.86; 86.85]	< 0.001	82.73	[61.54, 103.92]	< 0.001
Time						
Baseline	Reference			Reference		
Follow-Up	-0.43	[-1.52, 0.67]	0.443	1.65	[-1.46, 4.76]	0.299
Group of the child						
Control	Reference			Reference		
Intervention	0.64	[-0.85, 2.14]	0.400	1.71	[-2.92, 6.35]	0.469
Age of the child (in years)	-0.42	[-0.73, -0.11]	0.007	-0.81	[-2.10, 0.49]	0.223
Sex of the child						
Female	Reference			Reference		
Male	-1.73	[-3.11, -0.36]	0.014	-0.18	[-4.60, 4.23]	0.936
Educational level of the parent						
Low	Reference			Reference		
Medium	1.62	[-0.95, 4.19]	0.217	1.61	[-5.36, 8.58]	0.651
High	0.58	[-1.92, 3.08]	0.649	-1.97	[-8.62, 4.68]	0.562
HAP^a^	-0.96	[-4.36, 2.44]	0.579	0.53	[-9.83, 10.90]	0.920
SLD^a^	0.82	[-2.32, 3.97]	0.607	-0.08	[-9.77, 9.61]	0.987
NE^a^	-0.69	[-4.14, 2.75]	0.693	-1.52	[-12.26, 9.23]	0.782
CD^a^	-2.18	[-5.30, 0.94]	0.171	-1.47	[-11.52, 8.58]	0.774
Interaction: Time × Group	-0.55	[-2.11, 1.02]	0.493	-2.43	[-7.33, 2.46]	0.329
Variance Intercept	67.02			116.55		
AIC^b^	10440.35			2011.67		

^*a*^*HAP* head and abdominal pain, somatoform, *SLD* developmental disorder of speech and language, *NE* non-organic enuresis, *CD* conduct disorder

^*b*^*AIC* Akaike information criterion

Conducting this analysis with the KINDL subscale scores parent proxy-report version (Table 3), no effect between intervention and HRQoL was found. As for the KINDL overall score, older age was significantly associated with lower HRQoL levels for the subscales ‘*emotional well-being’* (-0.73 points; 95% CI [-1.08; -0.37], ‘*self-worth’* (-0.77 points; 95% CI [-1.17; -0.38] and ‘*school-related well-being’* (-0.67 points; 95% CI [-1.15; -0.18]. For the subscales ‘*self-worth*’, ‘*friends’,* and ‘*school-related well-being’* boys had on average lower HRQoL than girls (*‘self-worth’*: -1.96 points; 95% CI [-3.77; -0.16]; ‘*friends’*: -2.45 points; 95% CI [-4.26; -0.64]; *‘school-related well-being’*: -4.12 points; 95% CI [-6.3; -1.93]). For the subscale *‘physical well-being’* children diagnosed with *head and abdominal pain* (-5.55 points; 95% CI [-10.87; -0.23]) and for the subscale *‘friends’* children with *conduct disorders* (-4.56 points; 95% CI [-8.69; -0.42]) had lower HRQoL levels.

**Table 3:** Results of the linear mixed effects model with the KINDL subscale scores parent proxy-report as dependent variable controlled for time, age (in years), sex, educational level of the parents and diagnoses

*n* = 891	**Physical well-being**			**Emotional well-being**			**Self-worth**			**Well-being in the family**			**Well-being related to friends/peers**			**School-related well-being**		
	Est.^a^	95%-CI	*P*-value	Est	95%-CI	*P*-value	Est	95%-CI	*P*-value	Est	95%-CI	*P*-value	Est	95%-CI	*P*-value	Est	95%-CI	*P*-value
Intercept	80.71	[73.72; 87.70]	< 0.001	87.36	[82.02; 92.69]	< 0.001	78.97	[73.04; 84.91]	< 0.001	81.53	[75.86; 87.19]	< 0.001	84.02	[78.1; 89.94]	< 0.01	82.99	[75.87; 90.11]	< 0.001
Time																		
Baseline	Reference			Reference			Reference			Reference			Reference			Reference		
Follow-Up	0.11	[-1.96; 2.18]	0.920	-0.55	[-1.90; 0.81]	0.429	0.24	[-1.35; 1.82]	0.772	-0.55	[-2.07; 0.97]	0.475	-0.84	[-2.49; 0.80]	0.314	-0.97	[-2.82; 0.89]	0.308
Group of the child																		
Control	Reference			Reference			Reference			Reference			Reference			Reference		
Intervention	0.29	[-2.05; 2.64]	0.807	0.57	[-1.22; 2.37]	0.531	0.41	[-1.57; 2.39]	0.682	1.78	[-0.13; 3.69]	0.068	-0.53	[-2.51; 1.46]	0.603	1.7	[-0.68; 4.08]	0.162
Age of the child (in years)	-0.16	[-0.62; 0.31]	0.508	-0.73	[-1.08; -0.37]	< 0.001	-0.77	[-1.17; -0.38]	< 0.001	-0.37	[-0.75; 0]	0.053	0.05	[-0.34; 0.45]	0.793	-0.67	[-1.15; -0.18]	0.007
Sex of the child																		
Female	Reference			Reference			Reference			Reference			Reference			Reference		
Male	-0.42	[-2.55; 1.71]	0.698	-1.01	[-2.66; 0.63]	0.227	-1.96	[-3.77; -0.16]	0.033	-0.90	[-2.64; 0.85]	0.313	-2.45	[-4.26; -0.64]	0.008	-4.12	[-6.3; -1.93]	< 0.001
Educational level of the parent																		
Low	Reference			Reference			Reference			Reference			Reference			Reference		
Medium	3.58	[-0.47; 7.64]	0.083	2.22	[-0.87; 5.31]	0.160	2.55	[-0.86; 5.97]	0.143	-0.30	[-3.59; 2.99]	0.858	0.46	[-2.97; 3.89]	0.793	0.65	[-3.46; 4.75]	0.757
High	3.95	[0.01; 7.89]		1.13	[-1.88; 4.14]		1.91	[-1.41; 5.23]		-2.31	[-5.51; 0.9]		-1.34	[-4.68; 1.99]		-0.05	[-4.05; 3.94]	
HAP^b^	-5.55	[-10.87; -0.23]	0.041	-0.42	[-4.47; 3.63]	0.839	-0.63	[-5.16; 3.90]	0.785	3.72	[-0.58; 8.02]	0.090	-1.61	[-6.11; 2.89]	0.484	-0.56	[-5.93; 4.82]	0.839
SLD^b^	-0.96	[-5.86; 3.94]	0.702	1.79	[-1.95; 5.54]	0.347	-0.19	[-4.37; 3.99]	0.929	2.40	[-1.57; 6.38]	0.236	-1.66	[-5.81; 2.50]	0.434	2.95	[-2.01; 7.91]	0.243
NE^b^	-4.31	[-9.61; 0.98]	0.110	-0.62	[-4.74; 3.5]	0.769	0.13	[-4.41; 4.68]	0.954	-0.12	[-4.48; 4.24]	0.957	-0.9	[-5.43; 3.62]	0.696	1.83	[-3.6; 7.27]	0.509
CD^b^	-2.15	[-7.03; 2.74]	0.389	-0.57	[-4.29; 3.16]	0.7659	-2.55	[-6.72; 1.61]	0.229	-1.58	[-5.54; 2.39]	0.436	-4.56	[-8.69; -0.42]	0.031	-1.64	[-6.57; 3.29]	0.514
Interaction: Time × Group	-1.45	[-4.41; 1.51]	0.336	0.51	[-1.44; 2.45]	0.609	-2.02	[-4.3; 0.25]	0.082	-0.78	[-2.95; 1.39]	0.482	0.54	[-1.81; 2.89]	0.653	-0.71	[-3.36; 1.93]	0.597
Variance Intercept	121.21			93.02			103.86			98.24			99.70			157.66		
AIC^c^	12168.30			11230.51			11559.19			11482.33			11604.12			11834.10		

^*a*^*Est* Estimate

^*b*^*HAP* head and abdominal pain, somatoform, *SLD* developmental disorder of speech and language, *NE* non-organic enuresis, *CD* conduct disorder

^*c*^*AIC* Akaike information criterion

There was also no effect between intervention and HRQoL found, when conducting this analysis with the KINDL subscale scores children self-report version (Table 4). Only for the subscale ‘family’ higher age of the child was significantly associated with lower HRQoL levels (-2.16 points; 95% CI [-3.88; -0.45]). Children in the intervention group had significantly decreased levels of the subscale ‘self-worth’ over time.

**Table 4:** Results of the linear mixed effects model with the KINDL subscale scores children self-report as dependent variable controlled for time, age (in years), sex, educational level of the parents and diagnoses

*n* = 163	**Physical well-being**			**Emotional well-being**			**Self-worth**			**Well-being in the family**			**Well-being related to friends/peers**			**School-related well-being**		
	Est.^a^	95%-CI	*P*-value	Est	95%-CI	*P*-value	Est	95%-CI	*P*-value	Est	95%-CI	*P*-value	Est	95%-CI	*P*-value	Est	95%-CI	*P*-value
Intercept	90.62	[62.40; 118.83]	< 0.001	85.75	[60.86; 110.65]	< 0.001	65.02	[38.14; 91.91]	< 0.001	107.49	[79.39; 135.59]	< 0.001	61.01	[30.49; 91.53]	< 0.01	88.98	[60.90; 117.05]	< 0.001
Time																		
Baseline	Reference			Reference			Reference			Reference			Reference			Reference		
Follow-Up	-0.71	[-5.72; 4.29]	0.780	2.41	[-1.77; 6.59]	0.258	5.10	[0.78; 9.42]	0.021	-0.12	[-5.07; 4.83]	0.962	3.38	[-1.22; 7.98]	0.149	1.98	[-3.55; 7.50]	0.483
Group of the child																		
Control	Reference			Reference			Reference			Reference			Reference			Reference		
Intervention	-2.43	[-8.59; 3.72]	0.439	3.58	[-1.84; 9.00]	0.195	2.85	[-3.04; 8.74]	0.343	3.81	[-2.37; 9.98]	0.227	2.51	[-4.17; 9.18]	0.462	0.41	[-5.83; 6.65]	0.898
Age of the child (in years)	-1.45	[-3.17; 0.26]	0.097	-0.79	[-2.3; 0.73]	0.308	-0.30	[-1.94; 1.34]	0.721	-2.16	[-3.88; -0.45]	0.014	0.74	[-1.12; 2.61]	0.434	-1.24	[-2.95; 0.48]	0.158
Sex of the child																		
Female	Reference			Reference			Reference			Reference			Reference			Reference		
Male	4.68	[-1.17; 10.53]	0.117	-2.51	[-7.68; 2.65]	0.340	0.23	[-5.37; 5.82]	0.936	-4.13	[-9.97; 1.71]	0.166	-1.02	[-7.38; 5.34]	0.753	1.02	[-4.83; 6.86]	0.733
Educational level of the parent																		
Low	Reference			Reference			Reference			Reference			Reference			Reference		
Medium	2.54	[-6.20; 11.27]	0.569	4.12	[-3.61; 11.86]	0.296	0.33	[-8.47; 9.14]	0.941	1.94	[-7.21; 11.09]	0.678	2.03	[-8.00; 12.05]	0.692	-3.11	[-12.23; 6.01]	0.504
High	-0.25	[-8.49; 7.99]		-1.68	[-9; 5.63]		-2.83	[-11.21; 5.55]		0.00	[-8.69; 8.69]	1.000	-5.61	[-15.17; 3.95]		-4.46	[-13.08; 4.17]	
HAP^b^	-5.36	[-18.53; 7.82]	0.425	1.26	[-10.36; 12.88]	0.832	2.96	[-10.22; 16.15]	0.660	1.34	[-12.48; 15.17]	0.849	7.34	[-7.59; 22.28]	0.335	0.04	[-13.89; 13.96]	0.996
SLD^b^	-1.22	[-13.66; 11.22]	0.847	-0.48	[-11.45; 10.50]	0.932	3.02	[-9.30; 15.34]	0.631	-1.62	[-14.53; 11.29]	0.805	0.98	[-12.99; 14.94]	0.891	1.31	[-11.67; 14.30]	0.843
NE^b^	-4.76	[-17.69; 8.16]	0.470	-3.12	[-14.57; 8.33]	0.593	7.52	[-6.06; 21.11]	0.278	-4.46	[-18.60; 9.69]	0.537	0.16	[-15.30; 15.63]	0.983	0.73	[-13.38; 14.84]	0.919
CD^b^	0.79	[-11.89; 13.47]	0.903	-0.50	[-11.69; 10.68]	0.930	1.05	[-11.74; 13.84]	0.872	-2.47	[-15.88; 10.95]	0.719	1.12	[-13.36; 15.61]	0.879	-4.84	[-18.36; 8.67]	0.483
Interaction: Time × Group	0.72	[-7.14; 8.57]	0.858	-2.75	[-9.32; 3.82]	0.412	-8.48	[-15.27; -1.70]	0.014	-0.36	[-8.12; 7.41]	0.928	-0.97	[-8.20; 6.26]	0.792	-5.93	[-14.58; 2.73]	0.180
Variance Intercept	158.20			135.75			168.13			158.20			234.97			122.68		
AIC^c^	2208.18			2134.42			2146.79			2186.87			2197.44			2220.16		

^*a*^*Est* Estimate

^*b*^*HAP* head and abdominal pain, somatoform, *SLD* developmental disorder of speech and language, *NE* non-organic enuresis, *CD* conduct disorder

^*c*^*AIC* Akaike information criterion

### Parental health-related quality of life model

Results from the linear mixed effects models for the effect of the HC with parental HRQoL are shown in Table 5. The model was based on *n* = 1005 parents. There was no effect between the children´s participation in HC and parental HRQoL after adjusting for age, sex, and educational level of the parent who answered the questionnaire, as well as treatment, diagnosis group of the child, and children´s HRQoL. The VAS score increased significantly over time (2.59 points; CI [1.29; 3.88]) for both groups. Higher HRQoL of the child was significantly associated with higher HRQoL of their parents (0.36 points; CI [0.30; 0.42]).

**Table 5:** Results of the linear mixed effects model with the VAS score as dependent variable controlled for time, age (in years), sex, educational level of the parent who answered the questionnaire, and diagnosis and health-related quality of life of the child

	**VAS**^**a**^ (*n* = 1005)		
	Estimate	95%-CI	*P*-value
Intercept	56.07	[47.42, 64.72]	< 0.001
Time			
Baseline	Reference		
Follow-Up	2.59	[1.29; 3.88]	< 0.001
Group of the child			
Control	Reference		
Intervention	1.07	[-0.66; 2.80]	0.225
Age of the parent [years]	0.02	[-0.13; 0.17]	0.787
Sex of the parent			
female	Reference		
male	0.01	[-2.53; 2.56]	0.991
Educational level of the parent			
Low	Reference		
Medium	-0.74	[-3.23; 1.75]	0.561
High	-0.14	[-2.40; 2.69]	
Diagnosis group child^b^			
HAP	-0.95	[-4.93; 3.02]	0.638
SLD	-0.60	[-4.29; 3.10]	0.752
NE	-1.42	[-5.49; 2.65]	0.494
CD	-1.19	[-4.86; 2.48]	0.526
HRQoL child^c^	0.36	[0.30; 0.42]	< 0.001
Interaction: Time × Group	-1.72	[-3.59; 0.15]	0.071
AIC^d^	11,723.31		

^a^*VAS* Visual Analogue Scale to measure parental health-related quality of life

^*b*^*HAP* head and abdominal pain, somatoform, *SLD* developmental disorder of speech and language, *NE* non-organic enuresis, *CD* conduct disorder

^c^*HRQoL* Health-related quality of life

^*d*^*AIC* Akaike information criterion

## Discussion

We investigated a standardized primary care program for the management of children and adolescents with mental health problems (MHP) but could not detect any effects of the program on health-related quality of life (HRQoL) of children being treated by a pediatrician trained in the HC specifications or their parents. HRQoL was lower in older children and in boys when reported by proxy. Parental HRQoL improved significantly over time.

Arguably, the lack of observed change in children’s HRQoL may be attributed to the relatively high levels of their HRQoL at baseline. Although MHP can have considerable negative impact on HRQoL of children and their families [1, 2], HRQoL levels of our sample were comparable with the general population, (mean 76.8 for parent proxy-report, 72.6 for child self-report) [39]. Also, in contrast to other studies, HRQoL remained stable or increased from baseline to follow-up [50, 51]. We hypothesize that both intervention and control group of our sample were a positive selection of insures because they had already been enrolled in an unspecific prevention program offered by the statutory health insurance funds.

Also, we could observe a distinct middle-class bias in our study population with over half of the participants reporting a high socioeconomic status. This seems surprising at first sight, since there is evidence that MHP are more prevalent in families with low socioeconomic status [14, 52–58]. To give an example, parental educational status was associated with persistence and severity of conduct disorders [59].

It can be argued that the HC might be better suited for some MH diagnosis groups. In our study, diagnoses were unevenly distributed with more than half of the participants being affected with developmental disorder of speech and language (SLD) which is also the most common single MH diagnosis in children in Germany (25% of all MH diagnoses) [5]. Having an aligned therapy meeting the needs of the present SLD and the related conditions, such as hearing, neurological, motor, cognitive, social, and emotional disorders, requires comprehensive diagnostic, in particular phoniatrics and pediatric audiology [60]. These multidisciplinary and elaborated assessments are not covered by primary care [61]. Likewise, children with SLD will be referred to speech therapists [61] leaving no real possibility for action for the primary care physician.

The results of our study provide supporting evidence in line with literature that children’s HRQoL is lower with proceeding age in children and adolescents [14, 31, 39, 62]. Reasons could be challenges at school, puberty stage or limited leisure time. In boys, we observed lower levels in HRQoL, which is also consistent with literature showing stronger impairment for boys than for girls [63].

Past research found that MHP in children are associated with decreased HRQoL levels regarding physiological, psychological and functional aspects. In contrast to this, the present study has shown lower levels in HRQoL only for children with CD and only for the subscale ‘well-being with peers’. This finding is supported by studies in children with attention deficit hyperactivity disorder (ADHD).

Our findings are particularly important as they include a patient-centered approach. Furthermore, the results complement further qualitative and quantitative components of the PrimAQuO study as the HC is a complex intervention [64] and its components may pursue different goals simultaneously [23, 24, 65]. Comprehensive program evaluations are necessary for optimized care for children and adolescents with MHP in primary care.

Some limitations of our study should be noted. First, our results are based on a survey sample of parents and their children who agreed to complete an online questionnaire. However, self-selection bias can hardly be avoided in this kind of study. Second, we lack information on the time of enrollment of the child in the HC, the period of treatment in the HC, and the time between treatment in the HC and data collection, all three factors, which might affect HRQoL and might introduce recall bias. Unfortunately, we do not have any data about the exact date, when the intervention had been conducted and about the timeframe between consultations at a pediatrician’s practice and the completion of the questionnaire. The reason was that we identified eligible children based on billing data in the health insurance records. However, billing data was available with a delay of up to six months. Nevertheless, we belief that the HC does not have immediate effects on HRQoL and rather expect the change to happen over the time of the one year follow-up. Third, given the character of the study, there was no random allocation to groups and the diagnoses groups were not balanced. Yet, we used propensity scores to reduce the effect of selection bias and compensate that there was no randomization. We were only able to analyze children already enrolled in a prevention program, therefore, any comparison to usual care has to be considered with caution. Forth, we lack of information on pediatricians program fidelity and the ability to cluster results by pediatrician as we do not have identifying data. Lastly, there are some known limitations in the measurement of HRQoL in children with MHP under eight years of age, likewise, the use of proxy versions might only be an approximation of the child´s HRQoL [66].

## Conclusion

This study made an attempt to verify the positive impact of this program that was found in a qualitative study with parents and other stakeholders [23]. Also, implementation of the program was found to be cost-neutral, which indicates that enrolled children caused less health care costs while effects were similar to usual care [24]. Although we could not show any quantitative effects, the approach of the HC may still be valid and improve health care of children and adolescents with MHP and should be evaluated in a more general population.

### Supplementary Information

**Additional file 1.**

## Data Availability

The datasets used and/or analysed during the current study available from the corresponding author (VL) on reasonable request. All statistical analyses were carried out using RStudio (Version 4.0.3). All R codes are provided by VL on request. For ethical concerns all the data cannot be publicly shared.
