# Correction: Impact of a pediatric primary care health‑coaching program on change in health‑related quality of life in children with mental health problems: results of the PrimA‑QuO cohort study

**DOI:** 10.1186/s12875-023-02181-8

**Published:** 2023-10-19

**Authors:** Verena Loidl, Karina Hamacher, Martin Lang, Otto Laub, Lars Schwettmann, Eva Grill

**Affiliations:** 1 Institute for Medical Information Processing, Biometry, and Epidemiology (IBE), Faculty of Medicine, LMU Munich, Munich, Germany; 2 Pettenkofer School of Public Health, Munich, Germany; 3 BKK Vertragsarbeitsgemeinschaft Bayern, Munich, Germany; 4 PaedNetz Bayern e.V, Munich, Germany; 5 Berufsverband der Kinder- und Jugendärzte (BVKJ) e.V, Cologne, Germany; 6 Helmholtz Zentrum München (GmbH) ? German Research Center for Environmental Health, Institute of Health Economics and Health Care Management (IGM), Neuherberg, Germany; 7 Department of Economics, Martin Luther University Halle-Wittenberg, Halle (Saale), Germany; 8 Division of Health Economics, Department of Health Services Research, School of Medicine and Health Sciences, Carl von Ossietzky University of Oldenburg, Oldenburg, Germany; 9 German Centre for Vertigo and Balance Disorders, LMU University Hospital, LMU Munich, Munich, Germany; 10 Munich Center of Health Sciences, Ludwig-Maximilians-Universität München, Munich, Germany

**Correction: BMC Primary Care 24:182 (2023)**

10.1186/s12875-023-02119-0

Following publication of the original article [1], the author reported that the third affiliation of author Lars Schwettmann was missing. Missing affiliation is added as 8th affiliation in this correction article.
