# Supplementary material for: Impact of a pediatric primary care health-coaching program on change in health-related quality of life in children with mental health problems: results of the PrimA-QuO cohort study
Source: BMC Prim Care. 2023 Sep 8;24:182. doi: 10.1186/s12875-023-02119-0 (PMC10486116; doi:10.1186/s12875-023-02119-0)
Supplement: Supplementary file 1 — Additional file 1. [file 12875_2023_2119_MOESM1_ESM.docx]

**Supplementary Information**

**Impact of a pediatric primary care health-coaching program on change in health-related quality of life in children with mental health problems: Results of the PrimA-QuO cohort study**

Verena Loidl^1,2^, Karina Hamacher^3^, Martin Lang^4,5^, Otto Laub^5^, Lars Schwettmann^6,7^, Eva Grill^1,8,9^

Appendix A:

The STARKE KIDS (SK) program

The BKK program “STARKE KIDS” (SK) is a health promotion program offered by the “Betriebskrankenkassen” (BKK) in Germany. The program is offered to BKK-insured families free of charge and is available nationwide. It is part of the BKK's broader commitment to promoting health and wellbeing among its members and the wider community. Children have to be enrolled by their parents in this program. Thereafter, they are provided additional developmental check-ups for children and adolescents enrolled in the SK program.

Supplementary Tab 1: Addtional developmental check-ups for children and adolescents enrolled in the STARKE KIDS program.

| **No** | **Screening measures of the SK program** | **Period** |
| --- | --- | --- |
| **1** | Parent counselling | Newborn (U0) |
| **2** | BKK Baby check | 1^st^ to 5^th^ months |
| **3** | Eye examination I | 5^th^ to 14^th^ months |
| **4** | Speech assessment | 20^th^ to 27^th^ months (U7) |
| **5** | Eye examination II | 20^th^ to 50^th^ months |
| **6** | Speech assessment | 33^rd^ to 38^th^ months (U7a) |
| **7** | Neolexon app for children with articulation disorders | 3 to 7 years |
| **8** | BKK primary school checkup I | 7 to 8 years |
| **9** | BKK primary school checkup II | 9 to 10 years |
| **10** | Vaccination advice HPV vaccination |  |
| **11** | Depression screening | 12 to 17 years |
| **12** | Media addiction screening | 12 to 17 years |
| **13** | BKK youth checkup | 16 to 17 yers |

The HEALTH COACHING (HC)

The SK is the foundation of the Health Coaching (HC), which can be offered to children and adolescents with mental health problems enrolled in the SK program. In our study these children form the intervention group (members of BKK SK and HC); while all others (members of BKK and SK (enrolled or not) but **no HC**) served as controls (Fig. 1).


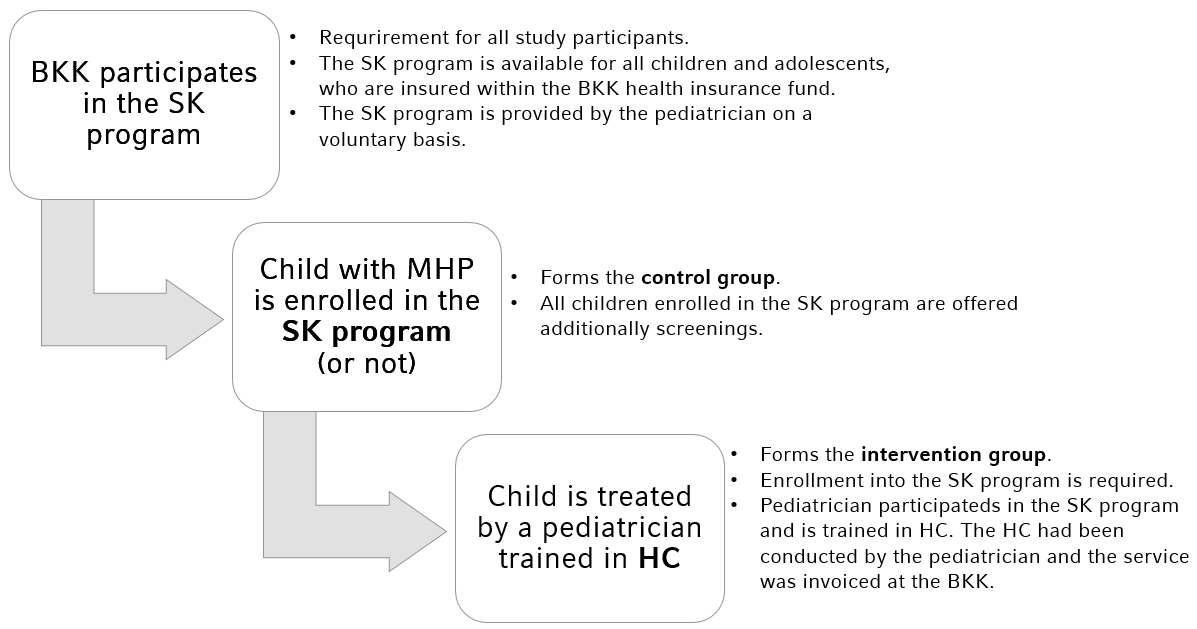


Supplementary Fig. 1 Overview of the allocation to the control (BKK STARKE KIDS (SK)) and the intervention group (SK + Health Coaching (HC)).

The HC has been developed by one of the major German statutory health insurance fund (*Betriebskrankenkassen Landesverband* (BKK-LV)) in collaboration with a professional association of paediatricians (*Berufsverband der Kinder- und Jugendärzte* (BVKJ e. V.)) in 2013. The HC has been predominantly implemented in Bavaria, one of the largest federal states of Germany, but has been available nationwide since October 2015. Up to now 96% (877/910) of participating pediatricians have been trained in HC. Compared to the end of this evaluation (30^th^ September 2020) 95% (832/874) had been trained in HC. According to the *Kassenärztliche Vereinigung Bayerns* (KVB) there are 1.286 pediatricians in Bavaria. Accordingly, most of the pediatricians completed the training in HC and thus, are able to treat children and adolescents with MPH according to the guidelines. Up to now approximately 36.000 children with MHP have been treated accordingly to the program. The HC includes a training concept for pediatricians, standardized action guidelines for 16 MHP, such as developmental disorders of speech and language, head and abdominal pain, enuresis, or conduct disorders. Within the HC, 15 euros per 10 minutes up to a cap of 180 minutes per child in addition to the statutory standard care are billable. The basic program principles are participation, patient orientation and strengthening of existing resources.

Appendix B:

Flowchart of participants’ progress through the phases of the PrimA-QuO study


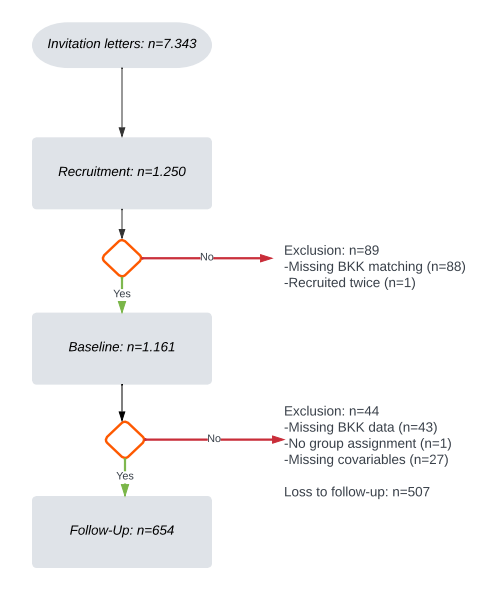


Supplementary Fig. 2: Flowchart of participants´ progress through the phases of the PrimA-QuO study.

Appendix C:

Questionnaires used in this study to measure health-related quality of life (HRQoL):

1. **KINDL^R^** (Kinder-Lebensqualitätsfragebogen)

Children´s mental health was assessed at baseline and follow-up using the German version of the KINDL^R^ Questionnaire.(1, 2) The child and adolescent self-assessment version was used for children aged 11 years or older. A parental report was available for younger children. The KINDL^R^ includes the six dimensions of quality of life, namely physical well-being, emotional well-being, self-esteem, family, friends and everyday functioning (school or nursery school/kindergarten) (Supplementary Tab 2). Each item provides answer on a five-point Likert scale ranging from “never” to “always” coded with values between 1 and 5. The higher values indicating "better" HRQoL ratings. The total HRQoL score was calculated for all 24 items. The item scores per dimension (and the total score) were added and transformed into values between 0 and 100 (total sum = total mean * 24; total score = ((total sum – 24)/96)*100).

Supplementary Tab 2: Structure of the sub-scales of the KINDL^R^ Questionnaire with its 24 items divided between six dimensions (with four items each) with reference to the past week.

| **Self-report version (11 to 17 year-olds)** | **Parents´ version (0 to 10 year-olds)** |
| --- | --- |
| **Physical Well-Being** | |
| 1. ... I felt ill  2. ... I was in pain  3. ... I was tired and worn-out  4. ... I felt strong and full of energy | 1. … my child felt ill  2. … my child had a headache or tummy-ache  3. ... my child was tired and worn-out  4. ... my child felt strong and full of energy |
| **Emotional Well-Being** | |
| 5. ... I had fun and laughed a lot  6. ... I was bored  7. ... I felt alone  8. ... I felt scared or unsure of myself | 5. ... my child had fun and laughed a lot  6. ... my child didn't feel much like doing anything  7. ... my child felt alone  8. ... my child felt scared or unsure of itself |
| **Self-Esteem** | |
| 9. ... I was proud of myself  10. ... I felt on top of the world  11. ... I felt pleased with myself  12. ... I had lots of good ideas | 9. ... my child was proud of himself  10. ... my child felt on top of the world  11. ... my child felt pleased with him-/ herself  12. ... my child had lots of good ideas |
| **Family** | |
| 13. ... I got on well with my parents  14. ... I felt fine at home  15. ... We quarrelled at home  16. ... I felt restricted by my parents | 13. ... my child got on well with us as parents  14. ... my child felt fine at home  15. ... we quarrelled at home  16. ... my child felt that I was bossing him/her around |
| **Friends** | |
| 17. ... I did things together with my friends  18. ... I was a "success" with my friends  19. ... I got along well with my friends  20. ... I felt different from other people | 17. ... my child did things together with friends  18. ... my child was liked by other kids  19. ... my child got along well with his/her friends  20. ... my child felt different from other children |
| **Everyday Functioning (School or Nursery School/Kindergarten)** | |
| 21. ... doing the schoolwork was easy  22. ... I found school interesting  23. ... I worried about my future  24. ... I worried about bad marks or grades | 21. ... my child easily coped with schoolwork  22. ... my child enjoyed the school lessons  23. ... my child worried about his future  24. ... my child was afraid of bad marks or grades |

1. **Visual Analogue Scale (VAS)**

To measure the parents´ health-related quality of life (HRQoL) we used the EQ-5D visual analogue scale (VAS)(3), which is a generic tool for patient-reported outcome measurement. The overall health state is rated on a scale of 0 to100 points. 0 points correspond to the worst possible health status, while 100 points correspond to the best possible health status.


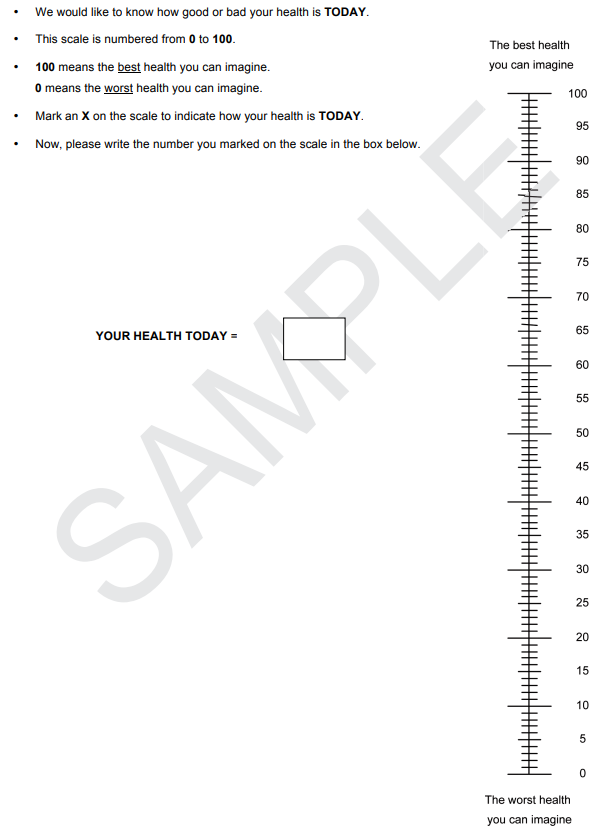


Supplementary Fig. 3: The VAS asks respondents to ‘mark an X on the scale to indicate how your health is TODAY’ and then to ‘write the number you marked on the scale in the box below’.(3)

References

1. Ravens-Sieberer U. Der Kindl-R Fragebogen zur Erfassung der gesundheitsbezogenen Lebensqualität bei Kindern und Jugendlichen - Revidierte Form. Göttingen: Hogrefe: Schumacher, J.; Klaiberg, A.; Brähler, E.; 2013.

2. Ravens-Sieberer U, Bullinger M. Assessing health-related quality of life in chronically ill children with the German KINDL: first psychometric and content analytical results. Quality of life research : an international journal of quality of life aspects of treatment, care and rehabilitation. 1998;7(5):399-407.

3. (Hrsg.) E. EQ-5D-User Guide. Basic information on how to use the EQ-5D-5L instrument. <https://euroqol.org/wp-content/uploads/2016/09/EQ-5D-5L_UserGuide_2015.pdf2015> [
